# The Double-Edged Sword: Naloxone-Induced Noncardiogenic Pulmonary Edema

**DOI:** 10.7759/cureus.41642

**Published:** 2023-07-10

**Authors:** Christopher Marsalisi, Hui Jun Guo, Pramod Reddy

**Affiliations:** 1 Internal Medicine, University of Florida College of Medicine – Jacksonville, Jacksonville, USA

**Keywords:** acute respiratory failure, acute respiratory failure with hypoxia, illicit fentanyl, pulmonary critical care, naloxone side effects, noncardiogenic pulmonary edema

## Abstract

Naloxone-induced noncardiogenic pulmonary edema (NCPE) is a scarcely reported side effect that can occur after naloxone administration. We present a case of a 46-year-old male who presented to the emergency department for further management of an opioid overdose, who developed acute hypoxic respiratory failure after several doses of naloxone. The rapid deterioration of the patient’s respiratory status required increased supplemental oxygen, with plane film radiography suggesting diffuse pulmonary edema. This case emphasizes the importance of understanding the significant side effects of a lifesaving drug and the implications they carry now that naloxone is available without prescription.

## Introduction

Naloxone is an opioid antagonist used for the rapid reversal of an acute opioid overdose. It works by binding to the μ-opioid receptors in the central nervous system, thereby reversing the respiratory depression and sedation caused by opioids [1]. Despite its efficacy and relatively safe drug profile, naloxone has been linked to several well-documented side effects, which coincide with withdrawal symptoms. These include tachycardia, seizures, dizziness, sweating, myalgias, and less commonly reported naloxone-induced noncardiogenic pulmonary edema (NCPE).

## Case presentation

A 46-year-old male with a past medical history of bilateral pulmonary emboli (on Eliquis), diabetes mellitus, and polysubstance use presented to the emergency department (ED) via emergency medical services (EMS) for the management of an acute opioid overdose. Prior to arriving in the ED, the patient was found unresponsive on the street and was noted to have strong femoral pulses, pinpoint pupils, and agonal respirations prompting administration of 4 mg of intranasal naloxone. The patient responded appropriately with an improvement in his mental status.

**Figure 1:**
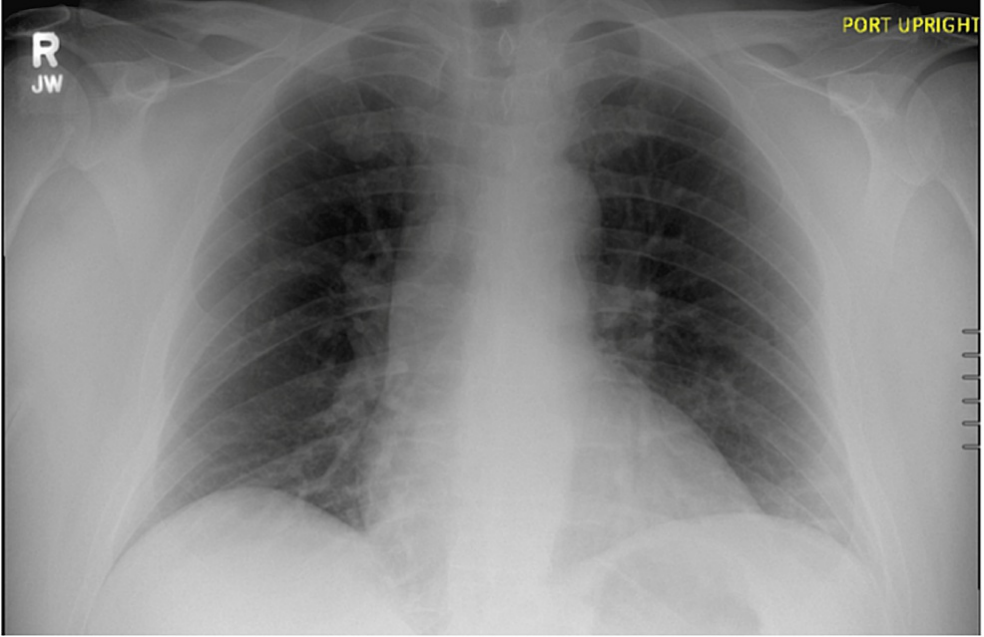
AP Chest X-Ray AP chest X-ray demonstrating no acute cardiopulmonary disease.

In the ED, the patient’s vitals were stable, with a blood pressure of 116/73, heart rate of 66, respiratory rate of 12, and pulse oximetry at 94% on room air. On physical examination, he was somnolent but arousable to voice and only oriented to person. The patient had an otherwise unremarkable physical exam with clear bilateral lung sounds and a cardiac exam with a regular rate and rhythm. Initial screening chest X-ray in the ED showed clear lung fields bilaterally and an unremarkable cardiac and mediastinal silhouette, lowering concern for acute cardiopulmonary involvement (Figure 1). Due to the patient’s concerning neurological exam, he received 0.4 mg of intramuscular (IM) naloxone in the ED without significant improvement, prompting a subsequent dose of 0.4 mg IM naloxone. Initial laboratory analysis was grossly unremarkable, with the exception of magnesium of 1.6, phosphorous of 1.4, and urine drug screen positive for fentanyl. 

**Figure 2:**
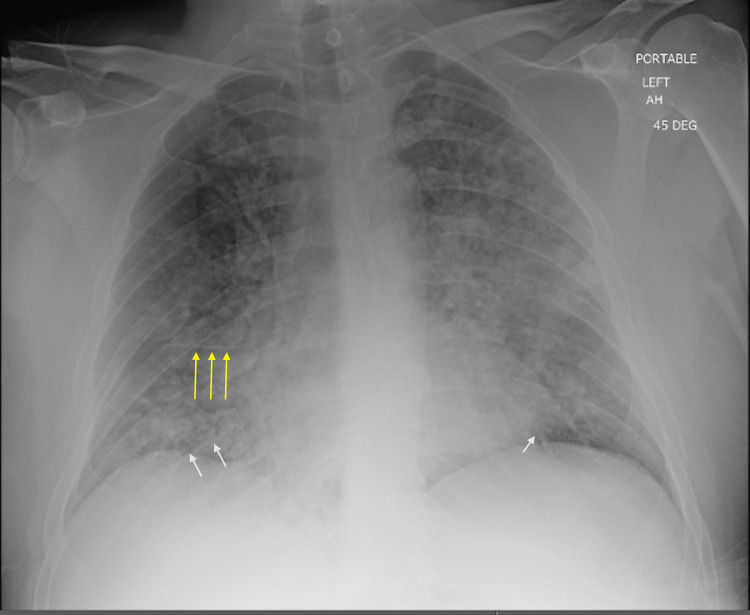
AP Chest X-Ray AP chest X-ray demonstrating diffuse interstitial opacities with thickening of the interlobular fissures, peribronchovascular cuffing, and blurring or indistinctness of the pulmonary vessel walls. Moreover, the white arrows demarcate Kerley C lines, and the yellow arrows demonstrate Kerley A lines. These septal lines, also known as Kerley lines, are seen when the interlobular septa in the pulmonary interstitium become prominent.

In the ED, the patient experienced several episodes of resedation due to the long half-life of fentanyl despite interval doses of 1 mg intravenous (IV) naloxone. After the fourth dose of naloxone, which was administered approximately one hour after initial patient contact in the ED, the patient became acutely hypoxic to 89% and responded poorly to up-titrated oxygen delivery through a nasal cannula (NC). He was transitioned to a high-flow NC (HFNC) 40L/60% due to persistent hypoxia. Interval plain film chest radiography demonstrated diffuse interstitial infiltrates (Figure 2). Point of care ultrasound (POCUS) at this time demonstrated a preserved ejection fraction (estimated left ventricular ejection fraction (LVEF) of 50-55%) based on endpoint septal separation (EPSS) with a fully collapsible inferior vena cava measured to be approximately 1.5 cm. Moreover, based on POCUS, the left ventricular function appeared preserved without any obvious wall motion abnormalities. Additional labs were drawn at the time of the acute change in the patient’s clinical status with NT-proBNP measured to be 150 pg/mL (0-125 pg/mL), zero-hour troponin <6 (negative delta troponin), and an electrocardiogram (EKG) at the time was normal sinus rhythm without acute ischemic changes.

**Figure 3:**
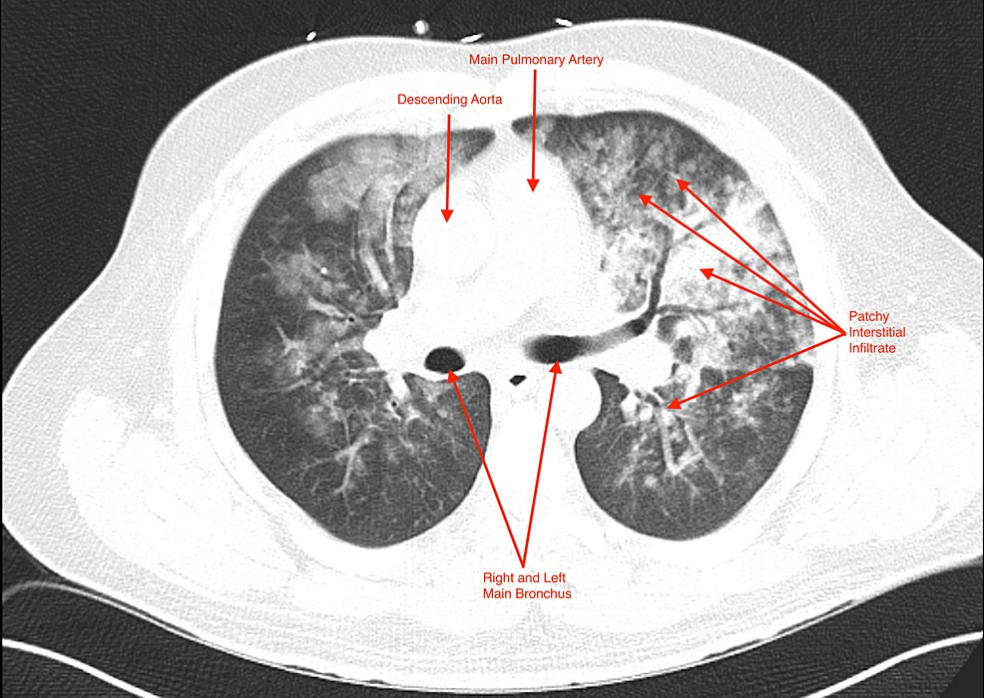
CT Chest Axial CT chest of the pulmonary vessel area demonstrating bilateral patchy opacities and diffuse tree-in-bud confluent ground-glass opacities.

**Figure 4:**
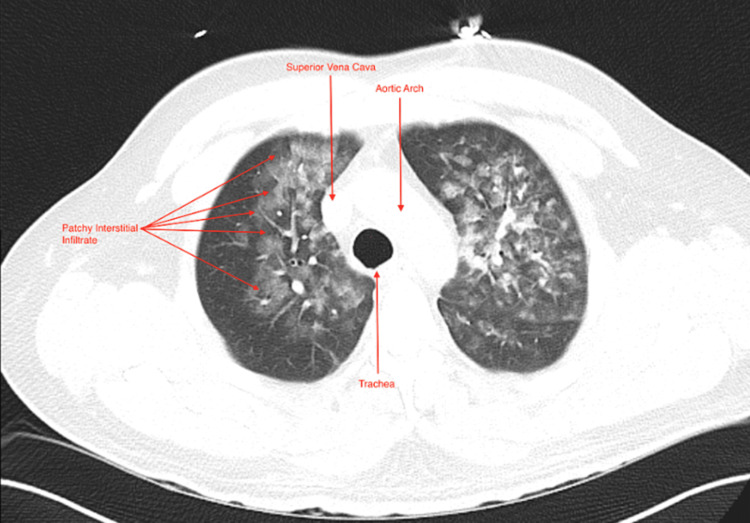
CT Chest Axial CT chest of the aortic arch area demonstrating bilateral patchy opacities and diffuse tree-in-bud confluent ground-glass opacities.

The patient’s clinical presentation was concerning for noncardiogenic pulmonary edema. He was subsequently managed with fluid restriction, nebulized albuterol, and HFNC. His interval clinical picture improved over the first 24 hours, and he was successfully weaned off HFNC to NC. A chest computerized tomography (CT) was obtained during this time, which demonstrated right lower lobe and left upper lobe patchy consolidative opacities with bilateral diffuse tree-in-bud confluent ground-glass opacities consistent with diffuse pulmonary edema (Figures 3-4). 

A transthoracic echocardiogram (TTE) was ordered during the patient's admission. TTE demonstrated a left ventricular ejection fraction of 55-60% with a normal diastolic filling pattern, thus effectively ruling out cardiogenic causes of pulmonary edema. Aspiration pneumonitis was also on the differential; however, the lack of fevers and leukocytosis makes the diagnosis less likely. The patient's respiratory status returned to baseline after three days of hospitalization, and he was safely discharged home after visiting the hospital's substance abuse program. 

## Discussion

Naloxone was first approved for the treatment of emergency opioid overdose by the Food and Drug Administration (FDA) in 1971. Despite its efficacy, deaths from opioid-related overdoses have continued to rise in recent years. A study from the National Institute on Drug Abuse in February of 2023 estimated a 26% increase in opioid-related deaths during the 11 years between 2010 and 2021 [2]. In recent years, there has been a growing recognition of the importance of making naloxone more widely available in the public sector to reduce opioid-related deaths. In efforts to liberate naloxone, the FDA recently authorized the distribution of this life-saving drug without a prescription in March of 2023. Although a success story, the wide availability of this medication comes with its risks, some of which are still not fully understood by specialists in the medical field.

One of the rare, life-threatening complications of naloxone is noncardiogenic pulmonary edema, which was suspected to be the cause of the presented patient's respiratory compromise. This side effect has been reported to occur at a rate of about 0.2-3.6%, and management of this complication involves supportive care and treatment of the underlying disease process [3,4]. Although the mechanism of naloxone-induced NCPE has not been fully elucidated, there is a well-supported theory that details the surge of catecholamines following naloxone administration as the culprit for pulmonary edema. This catecholamine surge is believed to result in pulmonary vasoconstriction and hydrostatic pressures, as well as an increased capillary permeability, resulting in a volume shift into the lung parenchyma [5,6].

Despite the rarity of this complication, there has been extensive research investigating the relationship between increased doses of naloxone and NCPE. In one of the most extensive studies to date published in the Annals of Emergency Medicine, investigators sought to understand the relationship between naloxone administration and pulmonary complications, such as pulmonary edema, aspiration pneumonia, and aspiration pneumonitis. The review of 1,831 patients treated with naloxone detailed that patients who received more than 4.4 mg of naloxone were 62% more likely to have pulmonary complications (odds ratio of 2.14; 95% confidence interval of 1.44-3.18). Despite this significant relationship, the study failed to demonstrate a clinically significant relationship between elevated naloxone doses (regardless of infusion or bolus approach) and pulmonary edema specifically [7].

In the presented case, the patient developed NCPE after receiving a total of 4.8 mg of naloxone via intranasal, IM, and IV routes. This case demonstrates the importance of being able to quickly identify when this complication occurs and the supportive treatment that should ensue thereafter. Moreover, this case report has significant implications when considering how widely available this medication has become given its recent approval for over-the-counter distribution. As of recent, naloxone can be purchased without a prescription for the treatment of acute opioid overdose. It is suggested that patients who purchase this medication over the counter should be made fully aware of the diverse side effects of this life-saving medication.

## Conclusions

Despite decades of nationally coordinated efforts to reduce the deleterious effects of the opioid epidemic, this crisis remains a significant cause of morbidity and mortality in the current day. Of the many remedies available in the medical armamentarium, naloxone remains the drug of choice for the management of acute opioid overdose. Although a relatively safe treatment, one of the most life-threatening complications of this medication is the development of pulmonary edema. Although previously believed to be dose-dependent, recent literature has debunked this hypothesis as NCPE has been documented to occur in cases of naloxone administration as low as 0.8 mg. In conclusion, this case emphasizes the importance of educating individuals in the medical as well as public sectors about this rare side effect. We hope that increased awareness of NCPE in the setting of naloxone use may improve treatment algorithms and reduce delayed management of this condition. 
